# Mechanical and structural optimization of flax fiber reinforced composites through controlled gamma irradiation

**DOI:** 10.1016/j.isci.2025.112531

**Published:** 2025-04-26

**Authors:** Madhu P, Sharath B N, Srinath M S, Pradeep S, Femiana Gapsari, Ari Wahjudi, Sanjay M R, Suchart Siengchin

**Affiliations:** 1 Department of Mechanical Engineering, Malnad College of Engineering (affiliated with Visvesvaraya Technological University, Belagavi), Hassan, Karnataka, India; 2 Mechanical Engineering Department, Faculty of Engineering, Brawijaya University, MT Haryono 167, Malang 65145, Indonesia; 3 Natural Composites Research Group Lab, Department of Materials and Production Engineering, The Sirindhorn International Thai-German Graduate School of Engineering (TGGS), King Mongkut’s University of Technology North Bangkok (KMUTNB), Bangkok 10800, Thailand

**Keywords:** Materials science, Materials synthesis, Composite materials

## Abstract

This study explores the effects of gamma irradiation on the mechanical and water absorption properties of flax fabric–reinforced epoxy composites for aerospace applications. Composites were fabricated using a manual lay-up technique and subjected to irradiation doses of 0, 100, 300, and 500 k rad. The laminate that was irradiated at 300 k rad (L3) had the best tensile, flexural, impact, and interlaminar shear strengths, and it also absorbed the least amount of water. Scanning electron microscopy showed better interfacial bonding and fewer defects, which proved that L3 had better structural integrity and durability. These results highlight the potential of flax-epoxy composites as environmentally sustainable, durable, and lightweight materials for aerospace components. Their ability to withstand extreme conditions makes them suitable for structural panels, protective enclosures, and insulation layers in space applications. Further studies can refine these composites by exploring hybrid reinforcements and advanced fabrication methods for improved performance under diverse environmental conditions.

## Highlights

• Gamma irradiation enhances tensile and flexural strength of flax/epoxy composites
• Optimal 300 k rad dose boosts ILSS and impact resistance in the composite laminate
• Laminate L3 shows least water absorption and best mechanical performance
• SEM reveals minimal defects and strong bonding in irradiated L3 composite

## Graphical abstract

**Figure:**
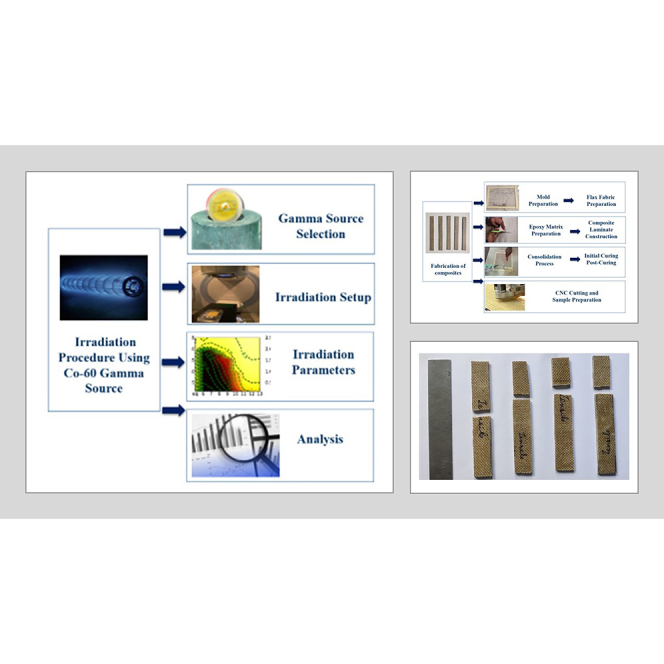

## Introduction

The aerospace industry is in pursuit of advanced lightweight, high-performance materials. Polymer composites reinforced with natural fibers are popular because of their strength-to-weight ratio, sustainability, and cost-effectiveness. This study investigates the impact of irradiation on the mechanical properties of flax fabric–reinforced epoxy composites, particularly in the context of aerospace engineering. Given spaceship components’ extreme environmental conditions, including radiation exposure, finding materials that can withstand such circumstances without compromising mechanical integrity is crucial.^1^^,^^2^^,^^3^ Epoxy has been chosen as the matrix material because of its remarkable adhesion and structural resilience, while flax fabric has been chosen as the reinforcing material because of its eco-friendly nature and promising mechanical properties. A substantial knowledge gap persists concerning the optimization of flax/epoxy composites for high-performance applications such as aeronautical engineering. Flax fibers have better mechanical properties and last longer, but it is still hard to get strong interfacial bonding with epoxy because they naturally attract water. Moreover, only a few research studies have explored the effects of gamma irradiation on flax/epoxy composites, particularly in terms of enhancing fiber-matrix adhesion, reducing void content, and enhancing mechanical performance in extreme environmental conditions. This work seeks to fill these gaps by examining flax/epoxy composites subjected to different gamma irradiation doses to enhance their characteristics for aeronautical applications. The incorporation of these materials is in line with the expansion of the need for aerospace composites that are both environmentally friendly and durable.^4^^,^^5^^,^^6^ Recent studies have further demonstrated the potential of incorporating filler particulates to improve composite properties. One example is Napier grass fiber–reinforced poly(lactic acid) composites with porcelain inserts. These composites had much higher tensile and flexural strengths because they had strong interfacial bonding, better antibacterial resistance, and less water absorption. These properties render these composites viable for sustainable building applications, automotive, and packaging.^7^ Similarly, adding porcelain fillers to basalt fiber–reinforced polymer composites made their thermal properties, such as their thermal stability and heat deflection temperature, much better. Therefore, experts deemed these composites suitable for thermal insulation in high-performance environments.^8^ The architecture of fiber substantially affects resin impregnation and the mechanical properties of natural fiber composites. A study on jute-polyester composites showed that the tensile and flexural properties got 54–124% better with the best twill architectures. These properties got 20–30% better with gamma irradiation, which was confirmed by FTIR, TGA, and SEM tests.^9^ Using gamma radiation to change flax fibers with methacrylamide grafting made them much more resistant to heat (by 50°C), but they kept their mechanical properties. The FTIR study showed a successful grafting yield of 55 wt %, while the SEM examination revealed no post-grafting damage, highlighting the method’s effectiveness in improving fiber performance.^10^ When flax fiber–reinforced PLA composites (20 wt %) were treated with triallyl isocyanurate (TAIC) and electron beam (EB) irradiation (40 kGy), their tensile strength went up by 20%. EB irradiation improved biodegradation by enzymes and composting, while adding TAIC made the material more resistant to these processes. This shows that crosslinking and branching play a dual role in the performance and durability of composites.^11^ Gamma radiation–assisted surface modification of flax fibers effectively created flax fiber–reinforced LLDPE composites, improving their compatibility with LLDPE. The better bonding between the surfaces led to much higher Young’s modulus, tensile strength, and thermal stability compared to pure LLDPE and flax composites that had not been changed, which was confirmed by SEM and dynamic mechanical analysis.^12^ The impact of gamma radiation on cotton, flax, and silk fabrics exhibited negligible alterations in mechanical qualities at low doses (up to 15 kGy). At elevated doses (100 kGy), tensile strength diminished by 26–33%. Radiation did not influence the fungal biodegradation of cotton and flax; nevertheless, silk showed heightened vulnerability to bacterial degradation under identical conditions.^13^ When hemp fibers and EB radiation were added to PCL composites, they showed higher flexural modulus but lower elongation at break and impact strength. Tensile strength fluctuated based on radiation dosage and fiber composition. Hemp fibers had a bigger effect on the mechanical properties than radiation. The composites had even fiber dispersion and good adhesion between surfaces.^14^ After being exposed to gamma rays at doses ranging from 50 to 1000 k rad, jute fabric-reinforced poly(caprolactone) biocomposites (50% jute) showed better strength in tensile, bending, and impact tests. The irradiation improved the physico-mechanical properties of the composites, and degradation characteristics and morphology were examined via SEM, validating structural enhancements.^15^ For the manufacture of composites, the manual lay-up approach was utilized. This method ensured perfect control over the orientation of the fibers and the distribution of the resin, both of which are essential for maintaining uniformity and performance.^3^^,^^6^ The composites were subjected to varying degrees of gamma radiation in order to simulate space-like conditions: no exposure, 100 K, 300 K, and 500 K. This study checks how well the composites work in these conditions by looking at their interlaminar shear strength (ILSS), tensile strength, flexural strength, impact strength, density, and void fraction.^16^ Furthermore, we evaluate the material’s durability in moisture-rich environments to determine its water absorption behavior, a critical factor in spacecraft longevity. Scanning electron microscopy (SEM) additionally offers a deeper understanding of the fracture mechanisms and the correlation between structural and mechanical behavior.^17^^,^^18^ This study investigates the flax/epoxy composites under extreme conditions, contributing to the development of environmentally sustainable materials for spacecraft. These findings will provide direction for enhancing composites of this kind, thereby boosting their dependability and endurance in the harsh environment of space missions. In the end, this research aims to assist in developing next-generation aircraft materials that satisfy both performance and sustainability criteria.^19^^,^^20^^,^^21^^,^^22^^,^^23^ The gamma irradiation has resulted in the development of composites that exhibit improved properties, rendering them promising candidates for aerospace applications. These applications include structural panels, protective enclosures, and insulation layers, where environmental sustainability, durability, and lightweight are important. This research enhances the comprehension of flax fabric–reinforced epoxy composites by methodically examining the impact of gamma irradiation at different doses (100, 300, and 500 k rad) on their mechanical, physical, and water absorption characteristics. Unlike most previous research that looked at untreated composites or hybrid fiber systems, this study shows how to use irradiation to improve overall performance for aerospace applications by increasing interfacial bonding, decreasing void content, and lowering void content. The results advance the creation of lightweight, durable, and environmentally friendly composite materials, providing significant insights into their performance under harsh conditions.

## Results and discussion

### Density and void fraction

Table 1 presents the theoretical and experimental densities, along with the void fraction, of the epoxy composites reinforced with flax fabric. The theoretical density of 1.2206 g/cc closely aligns with the experimental density of 1.212 g/cc. This near approximation suggests that the fabrication process was meticulously managed, resulting in a high level of precision in manufacturing. The recorded void percentage of 1.687% significantly falls below the widely recognized 5% threshold for aerospace-grade composites. This suggests that the fabricated composite is of exceptional quality. Composites with void percentages below 1% are generally regarded as optimal, while those with void percentages exceeding 5% are considered inadequate due to their compromised structural integrity and diminished water-resistant properties. The composite’s resistance to moisture penetration is adequate, as evidenced by the fact that the recorded void percentage in this study is slightly above the optimal level but still within acceptable limits. The low void percentage signifies the fabrication of the composite with minimal internal defects, a critical aspect for maintaining mechanical strength and durability in aerospace applications. Extreme environmental conditions, such as temperature fluctuations and radiation in space, significantly impact materials. By minimizing cavities, the material improves its mechanical performance under a variety of loading conditions and increases its resistance to environmental stresses.^24^^,^^25^^,^^26^ The results show that the epoxy composites reinforced with flax fabric are of high quality and work well for aerospace applications because they are close to having the right density and have a low percentage of space. The results substantiate the efficacy of the chosen fabrication techniques in achieving the composites' intended physical characteristics.

**Table 1:** Density and void fraction of prepared composites

Laminates	Theoretical density, *ρ*_*th*_ (g/cc)	Experimental density, *ρ*_*ex*_ (g/cc)	Void (%)
FFFF	1.2206	1.212	1.687%

### Mechanical properties

#### Tensile strength

The tensile characteristics of the laminates exhibited notable variations, underscoring the pivotal influence of gamma irradiation on improving mechanical performance. Among the laminates, laminate L3 demonstrated the maximum tensile strength of 35.23 MPa, markedly surpassing the other laminates. The improvement is because the right amount of gamma radiation (300 k rad) was used, which caused cross-linking in the epoxy matrix, better fiber-matrix adhesion, and less void content. These modifications led to enhanced load transfer efficiency and structural integrity. Conversely, lesser doses (L1 and L2) exhibited minimal enhancement owing to inadequate cross-linking, whereas higher doses (L4) resulted in matrix embrittlement, compromising tensile characteristics.

**Figure 1:**
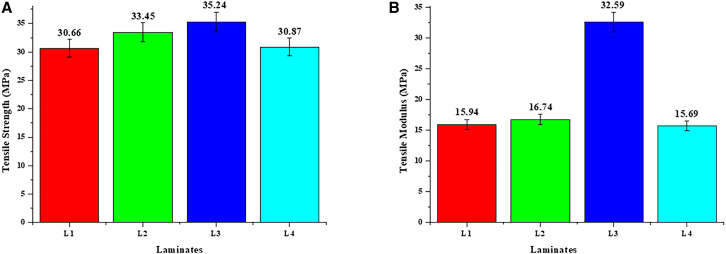
Tensile properties of Flax/Epoxy composites at different Gamma irradiation doses (A) Tensile strength: Shows the comparison of tensile strength for laminates subjected to different irradiation doses. (B) Tensile modulus: Demonstrates the variations in tensile modulus among the laminates.

The tensile modulus, a measure of rigidity, varied among laminates, with laminate L3 attaining the maximum modulus of 32.59 MPa. This indicates that laminate L3 exhibits enhanced resistance to deformation under tensile stress. The results demonstrate that the equilibrium between dosage and material reaction is essential for maximizing mechanical characteristics. Irradiation-induced cross-linking made the epoxy matrix more rigid, and getting rid of micro-voids made the composite stick together better. Figures 1A and 1B depict the tensile strength and modulus values, respectively, further substantiating the exceptional mechanical performance of laminate L3.

A detailed comparative assessment of tensile strength values from existing literature for natural fiber composites exposed to gamma or electron irradiation is provided in Table S1. These studies indicate that irradiation increases tensile strength, with optimal doses resulting in substantial enhancements in mechanical performance. The results for laminate L3 in this study align with these broader trends, where controlled irradiation improves fiber-matrix interaction and minimizes structural defects. Excessive doses frequently result in property degradation, as seen in higher-dose laminates and other research, highlighting the necessity for accurate dose optimization.

The results highlight the capability of laminate L3 for applications necessitating elevated tensile strength and rigidity, such as aerospace constructions. Its strong mechanical qualities, along with a minimal void content, guarantee longevity in harsh situations, rendering it appropriate for load-bearing components.^27^^,^^28^^,^^29^^,^^30^ This study emphasizes the necessity of optimizing manufacturing parameters to create advanced composites that satisfy rigorous technical requirements.

#### Flexural strength

**Figure 2:**
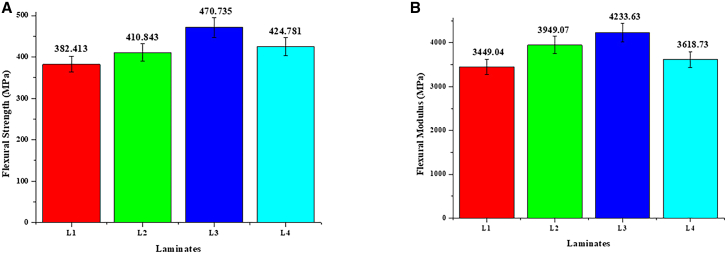
Flexural properties of Flax/Epoxy composites at different Gamma irradiation doses (A) Flexural strength: Depicts the flexural strength of laminates subjected to varying irradiation doses. (B) Flexural modulus: Highlights the differences in flexural modulus across the tested laminates.

The gamma irradiation method has a big effect on the laminates’' flexural strength, which is an important measure of how well they can handle bending forces. Among the laminates, laminate L3 displays the highest flexural strength at 470.735 MPa, indicating its superior ability to withstand bending stresses. This feature is crucial for aircraft applications, where structural components endure intricate and dynamic stress conditions. L3’s exceptional flexural performance highlights its suitability for applications necessitating materials that preserve integrity under complex pressures. Figures 2A and 2B depict the values of flexural strength and modulus, respectively.

The flexural modulus of laminate L3, recorded at 4223.63 MPa, underscores its resistance to deformation under bending stresses. The elevated modulus signifies that L3 can preserve its structural integrity and form, even under significant stress, rendering it a viable option for aerospace applications requiring both strength and rigidity. The reliable performance of L3 in both tensile and flexural tests underscores its versatility and durability in various loading conditions.

Multiple factors, including optimal laminate composition, greater fiber-matrix adhesion, and meticulous production methods, contribute to L3’s superior flexural capabilities. To get these qualities, the best gamma irradiation dose is 300 k rad. This increases the fiber-matrix interfacial connection, lowers the void content, and makes it easier for stress to be transferred when the material is bent. Regulated irradiation at this dosage reduces microstructural flaws, substantially enhancing the laminate’s resistance to bending stresses. Conversely, inadequate irradiation doses (L1 and L2) produced inferior fiber-matrix adhesion, whereas excessive doses (L4) caused matrix embrittlement, thereby reducing the flexural strength and modulus of the laminate.

A detailed comparative assessment of tensile strength values documented in the literature for natural fiber composites exposed to gamma or electron irradiation is presented in Table S2. These studies indicate that irradiation increases tensile strength, with optimal doses resulting in substantial enhancements in mechanical performance. The results for laminate L3 in this study align with these broader trends, where controlled irradiation improves fiber-matrix interaction and minimizes structural defects. Excessive doses frequently result in property degradation, as seen in higher-dose laminates and other research, highlighting the necessity for accurate dose optimization.

These findings provide significant insights into the design and production of advanced composites specifically engineered for aerospace applications. Laminate L3’s capacity to preserve elevated flexural strength and modulus while reducing structural imperfections highlights its appropriateness for essential components like structural panels and casings. The strength of L3 in tensile and flexural performance underscores its capability to endure the intricate pressures present in dynamic aircraft settings.^31^^,^^32^^,^^33^^,^^34^ This work highlights the importance of customizing material composition and processing parameters to achieve specific mechanical properties, thereby enhancing the development of high-performance composites for demanding engineering applications.

#### ILSS

**Figure 3:**
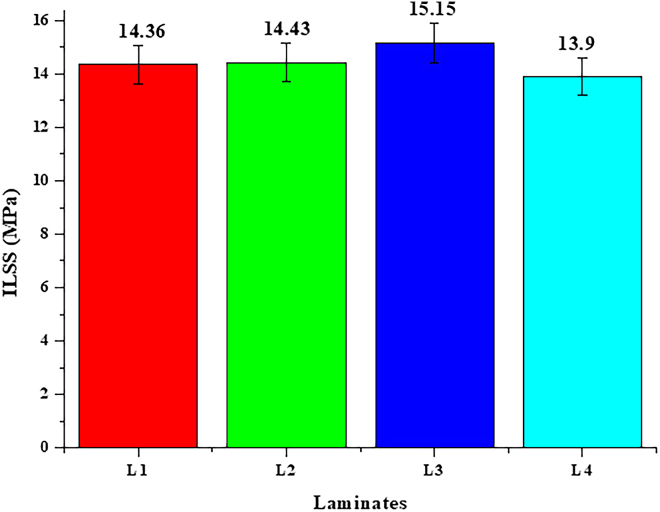
ILSS values of flax/epoxy composites at different irradiation doses Illustrates the interlaminar shear strength (ILSS) for laminates exposed to varying irradiation doses, emphasizing the superior performance of Laminate L3.

ILSS is a vital parameter that assesses a composite’s capacity to withstand shearing pressures between its layers, particularly significant for aeronautical applications. These applications expose materials to intricate and variable loading conditions, such as impact, torsion, and bending, where preserving structural integrity and longevity is essential. Figure 3 illustrates the ILSS values for the different laminates, emphasizing laminate L3’s exceptional performance with an ILSS of 15.15 MPa, the highest recorded among the tested laminates. This result shows that the composite layers stick together very well, which is a key part of keeping aircraft structures from delaminating when they are put under different kinds of stress.

The improved ILSS of laminate L3 highlights its ability to endure interlaminar stresses, positioning it as a robust option for aerospace components exposed to complex pressures during flight and maneuvering. The high ILSS value is because the 300 k rad gamma irradiation dose improved chemical bonding and decreased the amount of empty space at the fiber-matrix interface. The regulated irradiation enhanced the composite’s capacity to withstand interlaminar shear forces, hence enhancing mechanical stability and durability. On the other hand, lower doses (L1 and L2) did not create enough cross-linking for good bonding, but high doses (L4) damaged the matrix, making it less good at adhesion and shear resistance between surfaces.

The findings underscore the essential importance of optimal composition and processing parameters in attaining enhanced ILSS. The performance of laminate L3 shows how precision gamma irradiation can improve bonding properties, which are important for long-term safety and reliability in aircraft settings. These results make it clear how important it is to find the right balance in ILSS to keep structures strong and parts lasting longer, especially when there are problems with how they work. The enhanced bonding characteristics of laminate L3 indicate its potential as a protective barrier against delamination, a prevalent failure mode in composite materials; hence, it reinforces its appropriateness for aerospace applications.

A detailed comparative assessment of ILSS values for natural fiber composites documented in the literature is provided in Table S3. The data consistently demonstrate that controlled irradiation markedly improves ILSS, as evidenced by multiple investigations. Composites that are exposed to EBs or gamma irradiation usually have better fiber-matrix adhesion and less void content, which makes them more resistant to shear pressures between the layers. Excessive dosages may result in matrix deterioration and brittleness, underscoring the necessity for accurate dose adjustment. The ILSS values for laminate L3 correspond with these overarching trends, highlighting the necessity of customized production procedures to satisfy the rigorous requirements of aerospace applications.

These findings highlight the significance of ILSS in the design of high-performance composites, particularly in aeronautical engineering, where the meticulous balance of weight, strength, and durability is crucial. By comprehending and regulating fluctuations in ILSS, materials engineers can enhance the design and manufacturing of composites to satisfy the stringent demands of aircraft constructions. It is possible to use laminate L3 for applications that need better mechanical performance and long-lasting dependability because it has a high ILSS.^35^^,^^36^ This study emphasizes the possibility for enhancing composite design to develop materials that perform exceptionally under the intricate and dynamic conditions found in aircraft applications.

#### Impact strength

**Figure 4:**
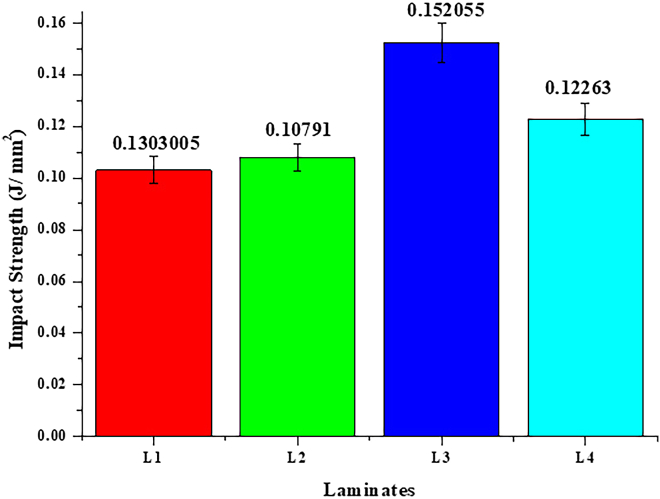
Impact strength of flax/epoxy composites at various irradiation doses Presents the impact strength of laminates subjected to different irradiation doses, showcasing Laminate L3’s enhanced resistance to sudden forces.

Impact strength, an essential indicator of a material’s capacity to withstand abrupt forces and avert fracture, demonstrated considerable discrepancies across the evaluated laminates. Laminate L3 exhibited the greatest impact strength at 0.152055 J/mm^2^, signifying its enhanced resistance to sudden forces relative to other laminates. Figure 4 presents the impact strength of the developed laminates, where L3 exhibited the highest energy absorption due to hybrid reinforcement. This improved capacity establishes L3 as a formidable contender for aerospace applications, where components frequently encounter dynamic and unforeseen stresses. The elevated impact strength of L3 highlights its superior energy absorption capability, an essential characteristic for guaranteeing material reliability and safety in aeronautical engineering.

Multiple factors influence the variations in impact strength seen among the laminates. Essential aspects encompass matrix dispersion, interfacial bonding, and fiber orientation within the composite. The best gamma radiation dose, 300 k rad, made L3 much more resistant to impact by increasing the cross-linking density in the epoxy matrix and decreasing the amount of empty space. These enhancements improved fiber-matrix adhesion, enabling the composite to dissipate energy more efficiently under abrupt loading circumstances. On the other hand, lower irradiation doses (L1 and L2) did not let enough energy escape because the fibers and matrix did not stick together well. On the other hand, too much irradiation (L4) made the matrix too crosslinked, which made it break easily and lose its ability to absorb energy.

The enhanced impact resistance of laminate L3 underscores the necessity of refining composite manufacturing procedures to get optimal mechanical properties. The results show that carefully controlling the amount of gamma irradiation and other fabrication parameters is necessary to make composites that are stronger against impact. This feature is especially beneficial for aircraft engineering applications, where structural components must withstand dynamic stresses and guarantee safety under unforeseen force circumstances. The enhanced performance of L3 indicates its suitability for aviation components, including structural panels, casings, and other load-bearing sections, where high-impact resistance is critical.

A detailed comparative assessment of impact strength values for natural fiber composites documented in the literature is provided in Table S4. The trends align with the findings of this study, demonstrating that controlled irradiation consistently improves impact resistance by enhancing fiber-matrix interactions and minimizing structural defects. Excessive dosages, however, frequently result in brittle behavior, underscoring the importance of accurate dose optimization.

This study’s results offer significant insights into the design and production of high-performance composites for rigorous engineering applications. By comprehending the determinants of impact strength, materials engineers can enhance manufacturing methods to create composites with superior energy absorption properties. Laminate L3 is perfect for aircraft applications because it is highly resistant to impact and quickly releases energy.^37^^,^^38^^,^^39^^,^^40^ This protects the structural integrity and durability of parts that are subjected to changing and unexpected pressures.

#### Water absorption behavior

To assess the water absorption characteristics of epoxy composites reinforced with flax fabric, specimens were submerged in distilled water, normal water, and saline for a duration of 20 days. This evaluation is indispensable for aerospace applications, as it offers critical insights into the material’s susceptibility to condensation in a variety of environmental conditions. Tables 2, 3, and 4 present the water absorption percentages for each laminate in the three distinct media. The laminates exhibit varying degrees of water absorption, with laminate L3 consistently exhibiting lower weight percentage increments than other laminates, as evidenced by the results. This diminished propensity to incorporate moisture implies that L3 laminate possesses exceptional resistance to water infiltration, which is advantageous for aerospace applications that prioritize material integrity and minimize weight gain.

**Table 2:** Water absorption percentage in distilled water

Laminates	Weight of the specimens before immersion (g)	Percentage increase in weights				
		Day 4	Day 8	Day 12	Day 16	Day 20
L-1	0.510	4.3	5.6	2.0	2.0	0.6
L-2	0.513	3.0	4.9	4.3	3.9	1.7
L-3	0.407	2.7	4.6	3.1	4.8	2.7
L-4	0.488	2.5	4.2	3.7	5.1	2.9

**Table 3:** Water absorption percentage in normal water

Laminates	Weight of the specimens before immersion (g)	Percentage increase in weights				
		Day 4	Day 8	Day 12	Day 16	Day 20
L-1	0.483	3.2	5.7	7.6	7.8	7.0
L-2	0.498	3.0	4.7	5.9	6.1	5.6
L-3	0.452	2.6	4.3	3.8	3.5	3.3
L-4	0.457	2.4	4.5	5.1	4.1	3.2

**Table 4:** Water absorption percentage in salt water

Laminates	Weight of the specimens before immersion (g)	Percentage increase in weights				
		Day 4	Day 8	Day 12	Day 16	Day 20
L-1	0.497	2.5	4.7	7.7	6.1	6.3
L-2	0.479	2.4	3.5	5.4	4.7	5.0
L-3	0.522	2.6	3.4	6.3	5.0	4.9
L-4	0.512	2.8	4.1	5.3	4.6	5.3

The lowest increase in weight percentage of L3 laminate in distilled water at all intervals indicated a lower susceptibility to moisture absorption. Laminate L1 also demonstrated resilience, registering the lowest percentage increase by Day 20. This provides additional evidence for the prospective applicability of L1 laminate in aerospace environments that necessitate minimal moisture absorption. All composites demonstrated a consistent pattern of water absorption under standard water conditions. Nevertheless, L3’s ability to demonstrate reduced weight percentage increases during the testing period underscores its potential as a suitable option for aerospace applications that are subject to typical weather conditions. The consistent performance of laminate L4 also indicates its resilience in environments where water exposure is a significant factor. Due to its corrosive properties, saltwater presented additional obstacles. In this environment, L3 laminate demonstrated the least weight gain, underscoring its capacity to effectively resist saline absorption. The performance of laminate L1 was also noteworthy, as it demonstrated significant resilience by Day 20 and suggested its suitability for use in scenarios that involved exposure to salinity. L4 demonstrated consistent resistance, which validated its suitability for use in marine or coastal aerospace environments.

Aerospace applications, where components frequently face a variety of weather and moisture conditions, require these composites’ water absorption characteristics. The findings suggest that aerospace structures are particularly well-suited to laminates with reduced water absorption tendencies, such as L3. It is essential to ensure that aerospace materials maintain their structural integrity and mechanical performance by minimizing water absorption. Excessive moisture intake can result in material degradation, increased weight, and compromised mechanical properties. The research underscores the significance of selecting and optimizing composite materials according to their water absorption properties. Laminates such as L3 exhibit significant potential as viable alternatives for aerospace components that necessitate weather-resistant properties and effective weight management. The results emphasize the necessity of additional improvements in composite design to enhance performance and longevity in difficult aerospace environments. Finally, a close study of how water absorbs characteristics gives useful information for choosing materials in the aerospace industry. This makes it easier to make composites that last longer and work better in a wider range of situations.^41^^,^^42^^,^^43^ The minimal water absorption of L3 underscores its appropriateness for aerospace applications, where moisture resistance is essential for preserving structural integrity and averting degradation in high-humidity conditions.^44^

#### SEM analysis

**Figure 5:**
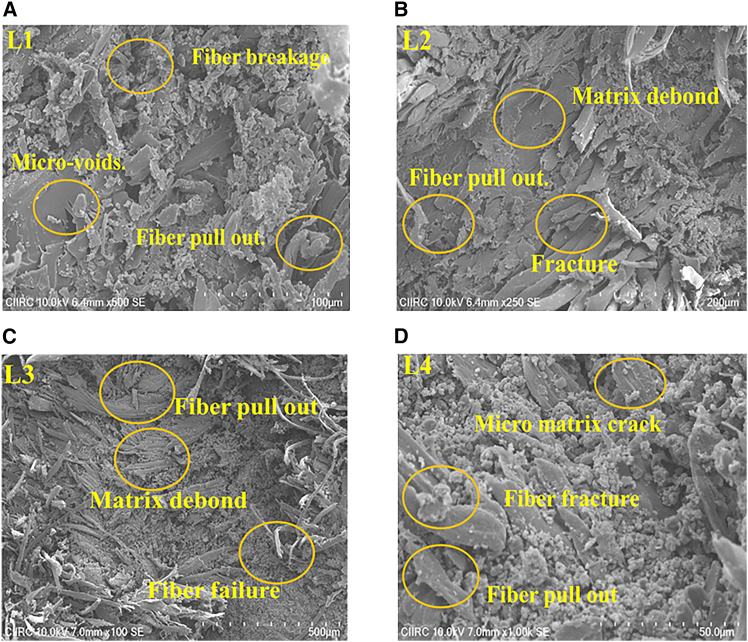
SEM micrographs of Flax/Epoxy composites showing fracture morphology after tensile testing (A) L1 laminate: Highlights minimal fiber pull-out and matrix separation. (B) L2 laminate: Displays fiber breakage and micro-voids. (C) L3 laminate: Demonstrates reduced fiber pull-out and superior interfacial bonding. (D) L4 laminate: Shows complex failure modes, including matrix cracking and fiber pull-out.

SEM analysis of fractured tensile samples critically illuminates the internal structure and failure mechanisms of the manufactured composites. Figures 5A–5D displays SEM micrographs of laminates L1, L2, L3, and L4.

Figure 5A shows that the L1 laminate has very little fiber detachment, very little matrix separation, and very little fiber fracture. This means that it is very resistant to tensile forces. There is less fiber pull-out and fewer micro-voids in L1, which shows that the interfacial bonding between the fiber and matrix is mostly intact. Micro-voids and clear fiber breakage can be seen in Figure 5B, which shows the L2 laminate. These features point to possible structural weaknesses and high-stress areas. This suggests that the L2 laminate may be more susceptible to internal stresses, which could result in premature failure during tensile loading. That being said, Figure 5C, which shows the L3 laminate, shows a big drop in fiber pull-out and matrix debonding, along with a smaller amount of fiber failure. This suggests that the superior interfacial adhesion and structural integrity of L3 enhance its mechanical performance. L3 is a prospective candidate for applications where durability is a critical requirement due to the well-bonded structure and the minimal evidence of separation. Figure 5D, which shows the L4 laminate, shows more complex ways that materials can fail, such as fiber pull-out, fiber breakage, and micro-matrix fissures. The fact that these things are present makes L4 less suitable for uses that need high structural reliability, since it could lead to delamination and weaker bonds.

The SEM analysis highlights the structural distinctions between the laminates, particularly the superior interfacial bonding and reduced failure features in L3. Aerospace applications subject materials to dynamic loading scenarios and extreme conditions, making L3 a highly promising candidate. L3’s exceptional structural integrity in aerospace environments suggests its long-term mechanical performance and structural reliability, as evidenced by minimal fiber pull-out and matrix debonding. Ensuring the durability of materials is essential for the successful completion of long-term missions in aerospace applications. The SEM results give us useful information about how different types of laminates fail in different ways. This helps us make better composite materials for very tough situations.^45^^,^^46^^,^^47^

### Conclusions

This study carefully looked at how the mechanical properties and water-absorbing abilities of epoxy composites strengthened with flax fabric changed when the irradiation intensity changed. When exposed to the right amount of radiation (300 k rad), laminate L3 showed improved properties such as higher tensile strength, flexural strength, ILSS, and resistance to impact. SEM tests proved that the increased interfacial bonding in L3 made the structure stronger and less likely to fail, including fiber pullout and matrix debonding. The water absorption test revealed that L3 had the lowest moisture uptake among the circumstances studied, highlighting its appropriateness for aerospace applications where weight reduction and durability are essential.

The findings demonstrate that irradiation-optimized flax fabric–reinforced epoxy composites, especially L3, exhibit considerable promise for aerospace engineering. These composites are appropriate for lightweight structural panels, protective casings, and insulating layers, providing radiation resistance, exceptional strength-to-weight ratios, and low water absorption. Moreover, the environmentally benign properties of flax-based composites correspond with the growing need for sustainable engineering materials, thereby mitigating environmental repercussions.

This study illustrates the feasibility of flax fabric–reinforced composites for aerospace applications; however, specific restrictions require attention. The study concentrated exclusively on flax/epoxy composites; investigating hybrid reinforcing or bio-based resin systems may further improve performance. Sophisticated techniques like vacuum bagging or resin infusion could enhance the existing fabrication method (manual layup with hydraulic pressing) and reduce void content. The study assessed merely three irradiation doses; exploring a wider spectrum and differing dosage rates may yield a more thorough comprehension. The lack of long-term environmental testing, including exposure to UV radiation, heat cycling, and microgravity, constrains understanding of endurance under extreme aerospace settings.

Subsequent research must tackle these constraints by refining material combinations and manufacturing methods to improve durability and performance under adverse conditions. Integrating hybrid reinforcement or bio-based resins may enhance the applicability of these composites. In addition to aerospace, the adaptability and sustainability of these materials render them viable options for the automotive, construction, and packaging sectors where both high performance and environmental factors are of paramount importance.

## Resource availability

### Lead contact

Further information and requests for resources and materials should be directed to and will be fulfilled by the Lead Contact, Dr. Madhu P (pm@mcehassan.ac.in).

### Materials availability

This study did not generate new unique reagents. All materials used in this study, including flax fibers and epoxy resin, were sourced commercially and are available from their respective manufacturers.

### Data and code availability

• Data: The data supporting the findings of this study are available within the manuscript and the supplemental information.
• Code: This study did not use any custom code or software.
• Other Items: Any additional information required is available upon reasonable request to the lead contact.

## Acknowledgments

We are grateful to the Faculty of Engineering, Brawijaya University, Dr. Hemaraju B.C., Department of Physics, and Dr. Yashas Gowda T.G., Department of Mechanical Engineering, Malnad College Engineering, Hassan, and KMUTNB for facilitating the initiation of collaboration between universities and engineering colleges.

The budget of this research was allocated by the National Science, Research and Innovation Fund (NSRF) (Fundamental Fund 2024) and King Mongkut's University of Technology North Bangkok (Project No. KMUTNB-FF-68-A-01).

## Author contributions

M.P., S.B.N., and S.M.S. conceptualized the study. Methodology was developed by M.P., S.B.N., and P.S., while the experimental investigation was carried out by S.B.N., P.S., F.G., and A.W. Formal analysis and interpretation of data were performed by S.B.N., P.S., and M.R.S. Resources were provided by M.P., M.R.S., and S.S. Data curation was handled by S.B.N. and P.S. The original draft of the manuscript was written by M.P., S.B.N., and P.S., and critically reviewed and edited by M.R.S., S.S., F.G., and A.W. Visualization was managed by S.B.N. and P.S. Supervision was provided by M.P., M.R.S., and S.S., while project administration was managed by M.P. and M.R.S. No external funding was acquired for this study.

## Declaration of interests

Dr. M.R.S. is a Guest Editor for the Special Issue on Polymeric Materials for Transportation Sector published in iScience. The editorial process for this manuscript was managed independently by another Editor, and M.R.S. was not involved in the decision-making process for this manuscript. The authors declare no competing interests. All authors have reviewed and approved the manuscript and certify that the submission is original and not under consideration elsewhere.

## STAR★Methods

### Key resources table

**Table:** 

REAGENT or RESOURCE	SOURCE	IDENTIFIER
**Other**		

Flax fabric	Local Supplier, India	Batch No. F2025
Epoxy resin (LY556)	Huntsman Advanced Materials	Batch No. 556A
Hardener (HY951)	Huntsman Advanced Materials	Batch No. 951B
Co-60 Gamma Irradiation Facility	BARC, Mumbai, India	Facility ID: BARC-Gamma-2025
Universal Testing Machine (Instron 3382)	Department of Mechanical Engineering, MCE	Machine ID: UT-3382
Scanning Electron Microscope (JEOL JSM-IT500)	Department of Mechanical Engineering, MCE	SEM Model: JSM-IT500

### Method details

#### Materials

The primary reinforcement material for the composite fabrication was woven flax fabric (thickness of 0.5 mm) with an areal density of 200 g/m^2^ and a density of 1.4 g/cm^3^, which was obtained from Go Green Products. Flax fibers, known for their high strength-to-weight ratio, biodegradability, and sustainability, serve as an eco-friendly alternative to composite materials. The aim of improving the environmental characteristics of the composites is consistent with the selection of flax fabric. The epoxy resin LY 556 (density 1.2 g/cm^3^) and HY951 hardener were combined in a 10:1 ratio to produce the composite matrix. Epoxy matrices are highly regarded for their exceptional adaptability, superior strength, and adhesion. Preserving the structural integrity of the composite is essential, and the combination of HY951 hardener and LY 556 epoxy creates a durable and resilient matrix. These materials were obtained from Herenba Instruments and Engineers in Tamil Nadu. The purpose of this study is to enhance the development of composites that are both environmentally sustainable and mechanically robust by meticulously selecting the epoxy system and flax fabric utilized. The selected materials are appropriate for applications in spacecraft components or other situations that necessitate resistance to external factors, such as radiation, due to their optimal balance of durability and performance.

#### Fabrication of composites

**Figure 6:**
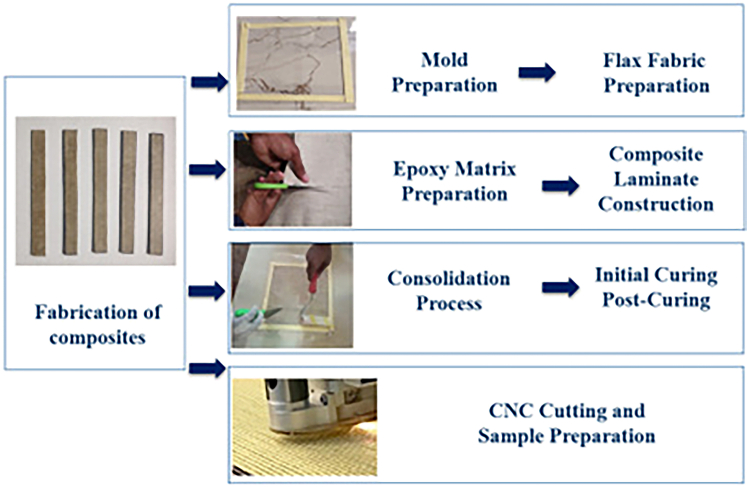
Fabrication of composites Illustrates the step-by-step process of fabricating flax fabric–reinforced epoxy composites using the hand lay-up technique. Includes key steps such as mold preparation, resin application, and curing.

**Figure 7:**
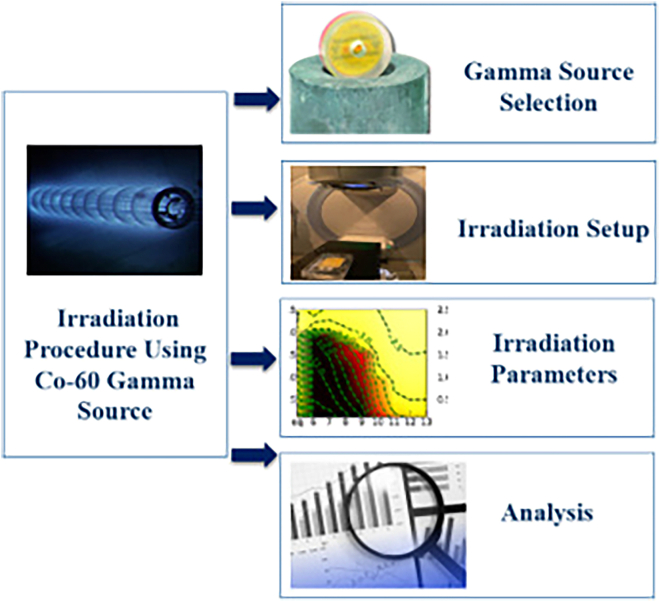
Irradiation procedure Depicts the irradiation setup utilizing a Co-60 gamma source with controlled dosage intervals. Highlights the experimental setup for uniform exposure.

The step-by-step fabrication process of the composite laminates using hand layup followed by compression moulding is shown in Figure 6. The hand lay-up technique was employed to guarantee the consistency and accuracy of the composite structure during the fabrication of the flax fabric-reinforced epoxy composites. The composite laminates were prepared using a mold with dimensions of 250 × 250 mm. The mold was meticulously cleansed and treated with a polyvinyl alcohol (PVA) mold release agent to ensure efficient release and prevent unwanted adhesion during the fabrication process, thereby achieving an ideal working surface. Subsequently, the flax fabrics were meticulously cut into layers in accordance with the necessary laminate layering sequence. This meticulous cutting process maintains the structural integrity and correct alignment of the flax fibers, ensuring uniform mechanical properties in the final composite. The composite laminates were constructed from four layers of flax fabric, weighing a total of 279 g, with a fiber weight fraction (W_f_) of 34.4% and a matrix weight fraction (W_m_) of 65.59%. The uniform stacking sequence (FFFF) guaranteed uniform fiber dispersion across the laminate. The epoxy matrix was prepared by mixing Epoxy LY 556 and HY951 hardener in a 10:1 weight ratio. The mixture was blended thoroughly using a mechanical stirrer at 300 rpm for 10 minutes to achieve uniformity. The epoxy mixture was then meticulously applied to each layer of the prepared flax fabric using a calibrated roller, ensuring complete saturation of the fibers and uniform resin distribution. This process not only facilitated a robust bond between the fibers and the matrix but also helped to expel any trapped air pockets, thereby minimizing voids. Following the resin application, the composite stack was subjected to a comprehensive consolidation process to enhance the fiber-matrix bond and further eliminate air pockets. The impregnated layers were carefully aligned and stacked inside the mold to maintain uniform thickness and proper fiber orientation. A rubber roller was used to apply gentle pressure, which ensured even resin distribution and expelled any residual air pockets. The stack was then placed in a hydraulic press, where a uniform pressure of 1.5 MPa was applied at room temperature for 24 hours. The final solidified composite laminates attained a uniform thickness of 3 mm. This step was crucial for achieving a compact, well-bonded composite structure with minimal void content. The laminates underwent dual stage curing to optimize their structural integrity. The composites were initially cured at room temperature for 24 hours to initiate polymerization. They were subsequently subjected to post-curing at 80°C for an additional 24 hours in a programmable hot air oven. This curing process ensured complete cross-linking of the resin matrix, enhancing the mechanical properties of the composites. A computer numerical control (CNC) cutting machine was utilized to guarantee accurate measurements and avert edge damage during the cutting process. The test samples were prepared in compliance with ASTM criteria for several tests. The samples were refined and deburred to guarantee smooth edges and precise testing. Meticulous attention was devoted to preserving consistent thickness among all specimens to guarantee reproducibility and consistency in test outcomes. Once fabricated, the composites were prepared for mechanical and physical testing under various irradiation conditions.^48^^,^^49^ Figure 7 illustrate the fabrication procedure of the composite laminates. The Equation 1^50^^,^^51^ below is used to calculate the fiber weight fraction and matrix weight fraction.

(Equation 1) $$W_{f}=w_{f}/\;\left(w_{f}+w_{m}\right)\;and\;W_{m}=w_{m}/\;\left(w_{f}+w_{m}\;\right)$$

where *w*_*f*_ indicates the weight of the flax fabric, *w*_*m*_ indicates the weight of the matrix, *W*_*f*_ is the weight fraction of fabric and *W*_*m*_ is the matrix weight fraction.

The fiber volume fraction that is *V*_*f*_ is calculated by using the Equation 2^52^^,^^53^ as shown below:

(Equation 2) $$V_{f}=\frac{\left(w_{f}/\;\rho_{f}\right)}{\left(w_{f}/\;\rho_{f}\right)+\left(w_{m}+\rho_{m}\right)}$$

The stacking sequence of the composite laminates is presented below, along with their corresponding irradiation doses:

**Table:** Stacking sequence of laminates

Laminates	Stacking sequence	Irradiation dosage
L1	F+F+F+F	Without irradiation
L2	F+F+F+F	100 K rad
L3	F+F+F+F	300 K rad
L4	F+F+F+F	500 K rad

F, Flax fabric.

The volume fraction and volume values for the composite laminates are detailed below:

**Table:** Volume fraction of composite laminate

Stacking sequence	Volume (mm^3^)			Volume fraction (%)	
	v_*c*_	v_*f*_	v_*m*_	V_*f*_	V_*m*_
FFFF	187500	24375	163125	14.63	85.36

#### Irradiation procedure

The irradiation procedure (Figure 7) for this investigation utilized a Co-60 gamma source, specifically the Model 650 No. 11R. An advanced capsule-type electromechanical mechanism, remotely controlled, ensures the reliability and precision of this gamma radiation delivery system. The system keeps the irradiation conditions stable and controlled by using a GBS-98 source to send the gamma beam instead of 36 double-encapsulated capsules.^54^^,^^55^ The Co-60 gamma source was selected due to its well-established efficacy and precision in delivering radiation. A dosage range of 100 to 500 k rad was chosen, with intervals of 200 k rad. This range was intended to encompass a wide range of prospective space exposure scenarios, thereby facilitating a thorough examination of the material’s behavior under varying radiation intensities. A dose rate of 232 K rad/hour was consistently sustained during the irradiation process, guaranteeing uniform exposure of all test samples. The technique was performed at ambient temperature and pressure, without alterations to the surrounding environment to maintain the integrity of the experimental apparatus. The dosage intervals were meticulously calculated to facilitate a comprehensive analysis of radiation effects, which simulated a diverse array of environmental conditions. The Co-60 system and the controlled loading mechanism of the GBS-98 source guaranteed precise and reproducible irradiation conditions. The use of a capsule-type system and remote-control functionality introduced an additional layer of reliability, crucial for the establishment of reliable experimental results.^16^^,^^56^^,^^57^^,^^58^ All test samples were utilized in their original form without any supplementary surface coating or treatment. This guaranteed that the outcomes precisely represented the material’s intrinsic reaction to gamma irradiation, unaltered by extrinsic influences. The study endeavored to offer a thorough understanding of the effects of varying radiation doses by subjecting the flax fabric-reinforced epoxy composites to a systematic irradiation procedure. The utilization of an advanced gamma source in this research enhances the reliability and credibility of the findings, allowing a comprehensive examination of the impact of varying radiation intensities on the mechanical and physical properties of these epoxy composites.

### Quantification and statistical analysis

#### Density and void fraction

To determine the fundamental physical properties of the flax fabric-reinforced epoxy composites that were prepared, a methodical evaluation of the density and void fraction was conducted by ASTM D2734-94.^59^ The Archimedes principle, which required determining the specimen’s weight in both air and a liquid medium, served as the basis for calculating the experimental density.

The experimental density (*ρ*_*ex*_) was calculated using the Equation 3^60^:

(Equation 3) $$\rho_{ex}=\frac{w_{a}}{w_{a}-w_{l}}\times \rho_{l}$$

Where the experimental density is denoted as *ρ*_*ex*_, the weight of the specimen in air is represented by *w*_*a*_, the weight in liquid is denoted by *w*_*l*_, and the density of the liquid is denoted by *ρ*_*l*_.

Concurrently, the theoretical density of each laminate was determined by conducting five trials and documenting the mean value. By applying the following Equation 4,^60^ the theoretical density was ascertained:

(Equation 4) $$\rho_{th}=\frac{100}{\left(\frac{W_{m}}{\rho_{m}}+\frac{W_{f}}{\rho_{f}}\right)}$$

Where the notation *ρ*_*m*_ denotes the density of the matrix phase, *W*_*m*_ represents the weight fraction percentage of the fabric, *W*_*f*_ indicates the weight fraction percentage of the fabric, and *ρ*_*f*_ signifies the density of the fabric.

Incorporating both theoretical and experimental densities, the void percentage was subsequently computed using Equation 5^60^:

(Equation 5) $$V_{v}=\frac{\rho_{th}-\rho_{ex}}{\rho_{th}}$$

Where the theoretical density is denoted as *ρ*_*th*_, while the experimental density is represented as *ρ*_*ex*_, with *V*_*v*_ representing the void percentage.

#### Tensile test

The tensile tests were performed according to ASTM D 3039 (254×25.4×3 mm)^61^ criteria using an Instron 33R Universal Testing Machine equipped with a 1000 kg load cell. Five trials were conducted for each laminate at a constant strain rate of 3 mm/min. Specimens were restrained throughout the test, and deflections caused by incremental loads were recorded until failure. The break load at failure was used to compute the composite’s ultimate strength. Detailed images of the tensile test specimens after testing are provided in Figure S1.

#### Flexural test

The flexural characteristics of epoxy composites reinforced with flax fabric were assessed using the ASTM D790-17 (90 × 10 × 3 mm)^62^ procedure. Using the three-point bending procedure, specimens were evaluated on the Instron 33R UTM machine at a constant strain rate of 1.15 mm/min with a span-to-thickness ratio of 16:1, corresponding to a span length of 48 mm. The load versus displacement and stress versus strain graphs provided valuable information regarding the modulus and flexural strength of the composites. Images of the flexural test specimens after testing are provided in Figure S2.

#### Interlaminar shear strength (ILSS) test

The ILSS of the prepared composites was determined using ASTM D2344-22 (60 × 10 × 3 mm).^63^ A short-beam shear test was conducted using a three-point bending fixture with a span-to-thickness ratio of 4:1. Stress-strain and load-displacement graphs were generated at a loading rate of 1.15 mm/min. The maximum load during testing was used to calculate the ILSS strength. Detailed images of the ILSS test specimens after testing are provided in Figure S3.

#### Impact test

The impact resistance of flax fabric-reinforced epoxy composites was evaluated following ASTM D256-10 (63×12.7×3 mm).^64^ An impact tester measured the joules of energy absorbed at fracture, providing a quantitative assessment of the material’s resistance to impact. Detailed images of the impact test specimens after testing are provided in Figure S4.

#### Water absorption behaviour

To examine the water absorption characteristics of flax fabric-reinforced epoxy composites, three distinct media were utilized: tap water, distilled water, and saline. Three specimens of each composite were submerged in its corresponding water medium at ambient temperature. The specimens were extracted, subsequently weighed, and cleansed using fresh tissue paper at designated intervals. Over twenty-eight days, readings were collected at consistent intervals. The dimensions of the test specimens were meticulously arranged to be 10×10×3 mm (ASTM D570-98).^65^ The water absorption test yields significant data regarding the composite material’s behavior in diverse water environments, thereby facilitating a thorough evaluation of its resilience and efficacy across a range of circumstances. By incorporating various water media, one can gain a more comprehensive comprehension of the material’s reaction to salinity and purity.

#### Morphological studies

To prepare broken specimens for tensile testing, sections less than 10×10 mm were cut. A coating of gold was applied to these sections to enhance conductivity and mitigate charging issues that may occur during imaging. The study employed SEM, more specifically a Hitachi SU 3500, to analyze the morphology of the fractured surface. Identifying voids, fiber pullouts, and fabric-matrix interfaces, and determining the adhesive properties between the reinforcement and matrix phases were the objectives. SEM examination provides visual representations of the flax fabric-reinforced epoxy composites' internal structure.^60^
