# Supplementary material for: Mechanical and structural optimization of flax fiber reinforced composites through controlled gamma irradiation
Source: iScience. 2025 Apr 26;28(7):112531. doi: 10.1016/j.isci.2025.112531 (PMC12269456; doi:10.1016/j.isci.2025.112531)
Supplement: Document S1. Figures S1–S4 [file mmc1.pdf]

iScience, Volume *28*

Supplemental information

Mechanical and structural optimization of flax fiber reinforced composites through controlled gamma irradiation

Madhu P, Sharath B N, Srinath M S, Pradeep S, Femiana Gapsari, Ari Wahjudi, Sanjay M R, and Suchart Siengchin

Figure S1: Composite tensile test specimens after testing. The specimens were prepared according to ASTM D 3039 standards (254×25.4×3 mm). The figure illustrates the post-failure condition of the specimens, highlighting the fractures and the quality of the laminate after tensile loading.

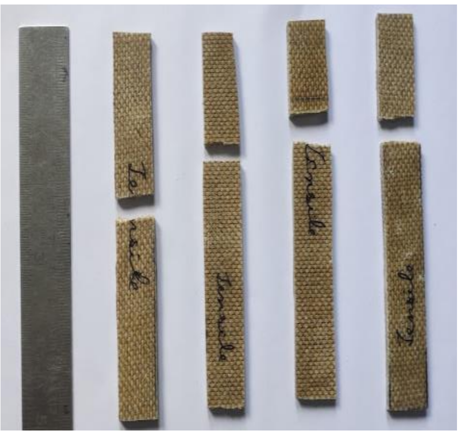

Figure S1: Tensile test specimens after testing

Figure S2: Composite flexural test specimens after testing. The specimens were prepared according to ASTM D790-17 standards (90×10×3 mm). This figure shows the post-testing condition of the specimens, highlighting the fracture behavior and deformation after undergoing three-point bending tests.

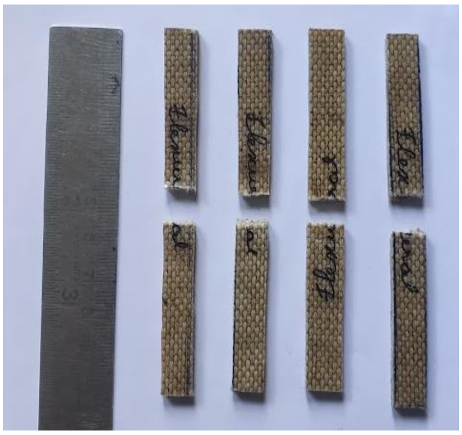

Figure S2: Flexural test specimens after testing

Figure S3: Composite ILSS test specimens after testing. The specimens were prepared in accordance with ASTM D2344-22 standards (60×10×3 mm). This figure shows the post-test

condition of the specimens, demonstrating failure modes and deformation after the short-beam shear test.

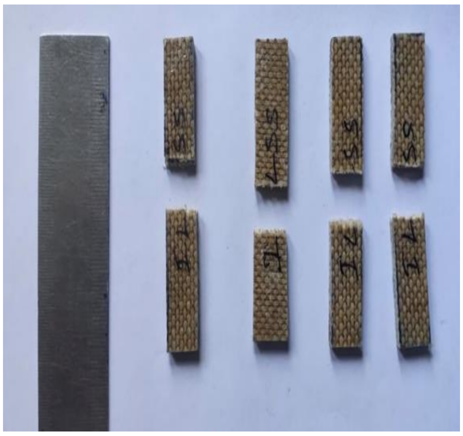

Figure S3: ILSS test specimens after testing

Figure S4: Composite impact test specimens after testing. The specimens were prepared following ASTM D256-10 standards (63×12.7×3 mm). This figure shows the condition of the specimens post-impact testing, demonstrating their fracture behavior and energy absorption capacity.

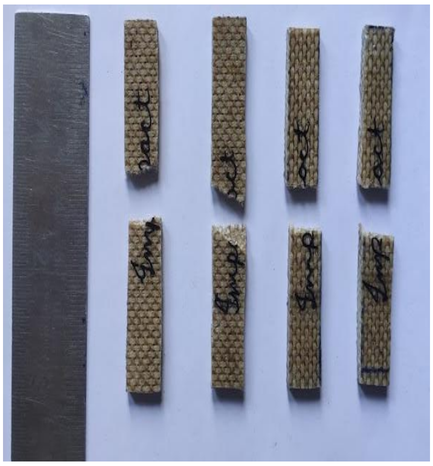

Figure S4: Impact test specimens after testing

Table S1: Comparative assessment of tensile strength of natural fiber composites exposed to gamma or electron irradiation. This table highlights the improvements achieved with controlled irradiation doses and contextualizes the results of this study within existing literature.

Table S1: Comparative Assessment of Tensile Strength of Natural Fiber Composites

**Fiber**	**Treatment**	**Irradiation Dose (kGy)**	**Tensile Strength (MPa)**	**Fabrication Method**	**Remarks**	**Ref.**
Flax	Gamma Irradiation	3	35.23	Hand Lay-Up	Optimal improvement at 3 kGy	Present Work
Hemp	Electron Beam Irradiation	10–90	40–55	Compression Molding	Mechanical improvement dependent on fiber ratio and dose	1
Flax	Gamma Radiation Grafting	1–4	No Significant Damage	Radiation Grafting	Maintained properties with enhanced thermal stability	2
Jute	Gamma Irradiation	0–50	48.74	Compression Molding	20–30% improvement for optimized composites	3
Carbon/Kevlar	Gamma Irradiation	1–5	215.36–246.28	Hand Lay-Up	Reduced with higher doses, observed transition to brittle fracture.	4

Table S2: Comparative assessment of flexural strength of natural fiber composites exposed to gamma or electron irradiation. This table highlights the flexural strength improvements achieved with controlled irradiation doses and contextualizes the results of this study within existing literature.

Table S2: Comparative Assessment of Flexural Strength of Natural Fiber Composites

**Fiber**	**Treatment**	**Irradiation Dose (kGy)**	**Flexural Strength (MPa)**	**Fabrication Method**	**Remarks**	**Ref.**
Flax	Gamma Irradiation	3	45.12	Hand Lay-Up	Optimal improvement at 3 kGy	Present Work
Hemp	Electron Beam Irradiation	10–90	50–60	Compression Molding	Significant increase with fiber content and radiation dose	1
Jute	Gamma Irradiation	0–50	96.56	Compression Molding	20–30% improvement with optimized architecture and dose	3
Carbon/Kevlar	Gamma Irradiation	1–5	578.49	Hand Lay-Up	Gradual reduction at higher doses due to brittle fracture	4

Table S3: Comparative assessment of ILSS values for natural fiber composites exposed to gamma or electron irradiation. This table highlights the improvements in interlaminar shear strength achieved with controlled irradiation doses and contextualizes the results of this study within existing literature.

Table S3: Comparative Assessment of ILSS of Natural Fiber Composites

**Fiber**	**Treatment**	**Irradiation Dose (kGy)**	**ILSS (MPa)**	**Fabrication Method**	**Remarks**	**Ref.**
Flax	Gamma Irradiation	3	8.92	Hand Lay-Up	Optimal improvement at 3 kGy	Present Work
Hemp	Electron Beam Irradiation	10–90	9.5–11.0	Compression Molding	Enhanced ILSS due to improved fiber-matrix adhesion	1
Jute	Gamma Irradiation	0–50	7.8–10.1	Compression Molding	Moderate improvement with optimized architecture and irradiation dose	3
Carbon/Kevlar	Gamma Irradiation	1–5	8.71–9.75	Hand Lay-Up	Gradual decrease at higher doses; transition to brittle behavior	4

Table S4: Comparative assessment of impact strength of natural fiber composites exposed to gamma or electron irradiation. This table highlights the improvements in energy absorption and mechanical performance achieved with optimized irradiation doses and contextualizes the results of this study within existing literature.

Table S4: Comparative Assessment of Impact Strength of Natural Fiber Composites

**Fiber**	**Treatment**	**Irradiation Dose (kGy)**	**Impact Strength (kJ/m²)**	**Fabrication Method**	**Remarks**	**Ref.**
Flax	Gamma Irradiation	3	14.25	Hand Lay-Up	Optimal improvement at 3 kGy	Present Work
Hemp	Electron Beam Irradiation	10–90	12.0–16.5	Compression Molding	Notable enhancement with higher fiber content and optimized dose	1
Jute	Gamma Irradiation	20–50	16.43	Compression Molding	20–30% improvement with optimized architecture and dose	3
Carbon/Kevlar	Gamma Irradiation	1–5	53.7	Hand Lay-Up	Gradual reduction at higher doses due to brittle behavior	4

References

1. Malinowski R, Raszkowska-Kaczor A, Moraczewski K, Głuszewski W, Krasinskyi V, Wedderburn L. The structure and mechanical properties of hemp fibers-reinforced poly

(ε-caprolactone) composites modified by electron beam irradiation. Applied Sciences. 2021 Jun 8;11(12):5317.

2. Agarwal R, Das P, Chowdhury SR. Gamma radiation-assisted functionalization of flax fibers for diversified applications. Journal of Radioanalytical and Nuclear Chemistry. 2024 Feb;333(2):687-94.
3. Azim AY, Alimuzzaman S, Sarker F. Optimizing the fabric architecture and effect of γ-radiation on the mechanical properties of jute fiber reinforced polyester composites. ACS omega. 2022 Mar 15;7(12):10127-36.
4. Sharath BN, TG YG, BC H, Madhu P. Gamma radiation-induced degradation of mechanical properties in Carbon/Kevlar hybrid epoxy composites for aerospace applications. Journal of Polymer Research. 2024 Dec;31(12):1-9.
